# Non-clinically trained facilitators’ experiences of remote psychosocial interventions for older adults with memory loss and their family carers

**DOI:** 10.1192/bjo.2023.558

**Published:** 2023-10-04

**Authors:** Philippa Renouf, Jessica Budgett, Danielle Wyman, Sara Banks, Michaela Poppe, Claudia Cooper

**Affiliations:** Division of Psychiatry, University College London, UK; and Centre for Psychiatry and Mental Health, Wolfson Institute of Population Health, Queen Mary University of London, UK; School of Psychology and Sport Science, Anglia Ruskin University, UK; Centre for Psychiatry and Mental Health, Wolfson Institute of Population Health, Queen Mary University of London, UK; Centre for Psychiatry and Mental Health, Wolfson Institute of Population Health, Queen Mary University of London, UK; and Memory Services, East London NHS Foundation Trust, UK

**Keywords:** Facilitation, psychosocial interventions, education and training, memory loss, remote delivery

## Abstract

**Background:**

Dementia is the seventh leading cause of global mortality, with cases increasing. Psychosocial interventions might help prevent dementia and improve quality of life. Although it is cost-effective for non-clinically trained staff to deliver these, concerns are raised and little is known about the resulting impact on staff, especially for remote interventions.

**Aims:**

To explore how non-clinically trained facilitators experienced delivering remote, one-to-one and group-based psychosocial interventions with older adults with memory loss and their family carers, under training and supervision.

**Method:**

We conducted a secondary thematic analysis of interviews with non-clinically trained facilitators, employed by universities, the National Health Service and third-sector organisations, who facilitated either of two manualised interventions: the APPLE-Tree group dementia prevention for people with mild memory loss or the NIDUS-Family one-to-one dyadic intervention for people living with dementia and their family carers.

**Results:**

The overarching theme of building confidence in developing therapeutic relationships was explained with subthemes that described the roles of positioning expertise (subtheme 1), developing clinical skills (subtheme 2), peer support (subtheme 3) in enabling this process and remote delivery as a potential barrier to it (subtheme 4).

**Conclusions:**

Non-clinically trained facilitators can have positive experiences delivering remote psychosocial interventions with older adults. Differences in life experience could compound initial fears of being ‘in at the deep end’ and ‘exposed’ as lacking expertise. Fears were allayed by experiencing positive therapeutic relationships and outcomes, and by growing confidence. For this to happen, appropriate training and supervision is needed, alongside accounting for the challenges of remote delivery.

As cases of dementia are projected to increase to 152 million worldwide by 2050,^1^ there is a clinical imperative to develop scalable psychosocial interventions, prevent dementia by addressing known modifiable risk factors^2,3^ and improve quality of life and independence of individuals living with dementia.^4^

## Non clinically trained facilitators

A cost-effective way to improve the scope and reach of such interventions is facilitation by non-clinically trained staff (without formal clinical qualifications) under training and supervision. The stepped care delivered by the National Health Service (NHS) Improving Access to Psychological Therapies (IAPT) uses this model, which is endorsed in the NHS Long Term Plan.^5^ Since its introduction in 2008, IAPT has increased the accessibility and reach of mental health services,^6^ although concerns have been raised about the impact of this work on non-clinically trained staff.^7^ Older adults (aged ≥65 years) are underrepresented in IAPT services,^5,8,9^ so it is important to consider how staff facilitators experience and are supported to deliver interventions to older clients. Because of the high community profile and outreach that third-sector organisations often have with older adults,^10^ they are also potentially well-placed to deliver interventions.

During the COVID-19 pandemic, many services moved to online remote delivery. Post-pandemic, remote delivery may provide another cost-effective, scalable way to deliver such interventions. Although online remote lifestyle interventions for older adults may be as effective as face-to-face delivery,^11^ little is known about facilitators’ experiences of remote delivery.

## Previous research

Kingstone et al^12^ explored the experiences of six third-sector support workers delivering an individual anxiety and depression intervention, face to face or via the telephone. Staff reported experiencing considerable anxiety, which was ameliorated by supervision, and their confidence increased with time. Amador et al^10^ report the experiences of three third-sector dementia support workers delivering a largely face-to-face, individual coping strategy intervention for family carers. Similarly, supervision and training increased their self-perceived capability and was a positive experience.

## This study

The current study is a secondary analysis of qualitative interviews originally conducted for the process evaluations of two psychosocial interventions. It includes the largest sample to date of community-based, non-clinically trained facilitators from university, NHS and third-sector sites. We aimed to explore how non-clinically trained facilitators experienced delivering a remote group-based intervention for older adults (aged ≥60 years) with objective or subjective cognitive decline; and a remote, one-to-one dyadic intervention for people living with dementia and their family carers.

## Method

### Ethics statement

The authors assert that all procedures contributing to this work comply with the ethical standards of the relevant national and institutional committees on human experimentation and with the Helsinki Declaration of 1975, as revised in 2008. All procedures involving human patients were approved by Camden and Kings Cross Research Ethics Committee (reference numbers 20/LO/0034 (ISRCTN17325135) and 19/LO/1667 (ISRCTN11425138)).

### Setting and sample

Participants (henceforth called facilitators) were staff who did not hold a formal clinical qualification and were working on either of two new remote psychosocial interventions: (a) the New Interventions for Independence in Dementia – Family (NIDUS-Family) study,^13^ a 1-year (six to eight manualised sessions) intervention delivered by video or telephone call to dyads (a person living with dementia and their family carer who took part as a pair, with the option of some one-on-one sessions), by one university-based facilitator (employed as research assistants); and (b) the Active Prevention in People at Risk of Dementia through Lifestyle, Behaviour Change and Technology to Build Resilience (APPLE-Tree) study,^3^ a six-month (ten fortnightly 1-h manualised sessions) group-based (six to eight person), video call intervention for people with mild memory loss, with an unstructured 30-min ‘tea break’ on the weeks in-between sessions and one-to-one fortnightly telephone calls, facilitated by two staff, usually one university-based facilitator (research assistant) and one NHS or third-sector staff.

Facilitators were employed by either university, NHS or third-sector organisations, and all were paid for their time. Intervention training was led by clinical psychologists, who taught clinical skills including active listening; opening and closing conversations; using supervision effectively; how to work in relational, personhood-upholding ways with people living with dementia; and considerations specific to remote delivery. Session delivery was practised by role-play to develop familiarity with the skills, session content and to troubleshoot potential scenarios. Facilitators were assessed by role-play before delivering the first intervention session, attended fortnightly group supervision with a clinical psychologist every fortnight and could access individual support.

We purposely selected non-clinical facilitators for maximum diversity across interventions, employer (university/NHS/third-sector) and gender. In total, NIDUS-Family was delivered by 11 university-employed non-clinical facilitators across two institutions. At time of writing, APPLE-Tree had been delivered by ten university-employed non-clinical facilitators (of whom three also facilitated NIDUS-Family), three NHS facilitators and nine third-sector facilitators, recruited through the teams’ networks and collaborating organisations, including Age UK, and various smaller third-sector organisations throughout London and South-East England.

### Data collection

Facilitators were invited to complete one semi-structured interview that used a topic guide (see Supplementary Appendices 1 and 2 available at https://doi.org/10.1192/bjo.2023.558) designed for the primary purpose of process evaluation, and focused on individuals’ experiences of delivering the interventions. Interviews were conducted by researchers who were trained and experienced in qualitative interview methods: either D.W., who did not facilitate the interventions, or by a university-employed researcher from a different team, to reduce risk of bias.

Written informed consent was obtained. Interviews were conducted on Zoom video-conferencing software (https://zoom.us/download), audio-recorded, transcribed verbatim and then anonymised before analysis.

### Data analysis

Data were analysed on NVivo version 20 for Windows (QSR International Pty Ltd., https://www.qsrinternational.com/nvivo-qualitative-data-analysis-software/home), following Braun and Clarke's^14^ six stages of reflexive thematic analysis (RTA). This methodology suited our aims to discover similarities and differences across the interviews, to build a deep understanding of the facilitators’ experiences.^15^ RTA acknowledges that the researcher's position and experiences are unavoidable and integral to the research (see ‘Reflexivity’ below).

An inductive coding framework based on line-by-line coding was used to allow facilitator experience to dictate the coding process. P.R. familiarised herself with the transcripts and identified sections relevant to the research aim (above). She coded all transcripts, developing broad codes based on semantic meaning, by grouping quotes based on content and keywords. She then reviewed these, considering latent meanings. She divided broad codes into subcodes, iteratively renaming and reorganising these in consultation with the author group. J.B. read 10% of transcripts and D.W. second coded 30% of transcripts to support exploration of emerging themes. P.R., C.C., J.B. and D.W. met multiple times to discuss interpretation, semantic and latent meaning, key themes and discrepancies. Our coding and thematic development explored and reported different interpretations where these arose in the group, considering how these might reflect different positions and experiences of facilitators (e.g. NHS or academic staff, age). A framework was agreed, which P.R. applied to all transcripts. Themes were iteratively developed, defined, named and presented below.

### Reflexivity

All co-authors acknowledge their positionality as ‘insider researchers’.^16^ P.R. has direct experience as a non-clinically trained facilitator for APPLE-Tree. The supervisory team (C.C., J.B. and M.P.) either line manage and/or advise non-clinically trained facilitators. S.B. and C.C. provided clinical supervision and C.C. is Principal Investigator for both studies. D.W. works on the process evaluation for the NIDUS-Family study and does not facilitate interventions or provide supervision/training.

The direct experience some researchers had both facilitating and training/supervising facilitators has implications for the analysis and interpretation of this data. Although we are aware this could create bias, ensuring interviews were not conducted by line managers, supervisors or the lead author (P.R.) aimed to reduce this, and we believe the positionality of the team provides richness to the understanding.

## Results

### Sample characteristics

Five researchers (including co-authors M.P., D.W. and J.B.) interviewed 17 out of 26 (65%) facilitators between September 2020 and October 2022. Interviews lasted on average 58 min. Table 1 shows facilitators’ characteristics.

**Table 1:** Demographic characteristics of facilitators

	Number of facilitators
Age, years	
18–25	5
26–30	8
≥31	4
Gender	
Male	3
Female	14
Sector	
Research assistant, university	12
Assistant psychologist, National Health Service	2
Charity worker, third sector	3
Number of intervention groups/dyads delivered to	
0–5	11
6–10	1
11–15	0
16–20	1
≥21	4
Years working with older adults with memory loss and their carers	
0–5	14
≥6	3
Years facilitating groups	
0–5	14
≥6	3

### Qualitative analysis findings

We identified one overarching theme of non-clinically trained facilitators building and maintaining therapeutic relationships. Facilitators initially lacked confidence in their capabilities to undertake this work. Some described a sense of ‘dread’ (third-sector facilitator 1, APPLE-Tree study) and felt ‘lost´ (university facilitator 6, NIDUS-Family study). All described some trepidation on beginning their role. One spoke of the ‘scale of the task’ (university facilitator 1, APPLE-Tree study) of effectively delivering the wide variety of intervention topics.

‘I mean it was a little bit nerve wracking and just because it was the first one, I think I was so lucky my first dyad was so lovely’ (university facilitator 1, NIDUS-Family study).

Named causes of anxiety were: time-keeping (sessions that ‘run over in time’) (university facilitator 4, NIDUS-Family study), facilitating positive group dynamics and avoiding ‘awkward moments’ (NHS facilitator 1, APPLE-Tree study), the uncertainty of client responses and clients who are ‘difficult to engage’ (university facilitator 5, APPLE-Tree study), ‘tricky questions’ (university facilitator 4, APPLE-Tree study) and not being ‘knowledgeable’ enough about more specialised content such as diabetes and blood pressure (university facilitator 1, APPLE-Tree study). Facilitators initially placed responsibility on themselves to be expert in these areas:

‘So what I was saying before was about me being too anxious and also me not having enough confidence in myself and also in the booklets themselves, and the groups being run properly’ (university facilitator 1, APPLE-Tree study).

This quote shows how for some, there were also concerns about whether the intervention would be sufficient for the task.

Across all accounts, these anxieties were at least partially allayed through positioning expertise (subtheme 1), building clinical skills (subtheme 2) and peer support (subtheme 3), whereas remote delivery could be a barrier to developing therapeutic relationships and confidence (subtheme 4).

#### Subtheme 1: positioning expertise

For most facilitators, negotiating therapeutic relationships involved initial discomfort in feeling positioned as an expert, by clients and self-expectations. This was largely abated by seeing positive results from their work.

Narratives illustrate that gaps in age and life experience between most facilitators and clients were keenly felt, with worries that material could ‘come over as quite patronising’ (NHS facilitator 2, APPLE-Tree study). One facilitator reflected that it was easier for positive therapeutic relationships to develop with clients ‘closer in age’ to them, in this example with a younger family carer:

‘We very much did have a laugh, we would joke and we got on … it was … a lot more informal and it was just more of a chat and we really did have a laugh about things … but those that were a bit older or … it did often feel different’ (university facilitator 3, NIDUS-Family study).

Where more difficult dynamics formed, facilitators often considered how age differences influenced this. One facilitator described feelings of being the client's ‘grandkid’ (university facilitator 3, NIDUS-Family study):

‘[they] felt very much like [the client] was a teacher and [the client] was telling me off’ (university facilitator 3, NIDUS-Family study).

Facilitators also noted an age gap between third-sector facilitators, who were usually older than those employed by universities or the NHS, who were typically in the first few years of their working lives. One older third-sector facilitator wondered about the capabilities of younger facilitators:

‘If they're young and they don't have any idea, how can they give advice to people when … they're not trainee doctors or nurses, so they might need, like with all the subjects, the clinical part, they definitely need a little bit of a chat to sort of let them know that diabetes is a very serious condition’ (third-sector facilitator 1, APPLE-Tree study).

A university facilitator felt that having less life experience meant she would be less capable than an older third-sector facilitator:

‘And I think it worked great with [co-facilitator] because [co-facilitator] has done so much in her life. She's very knowledgeable, she knows a lot about diabetes. She knows a lot about how GPs [general practices] work. [Third-sector facilitator] as well, she works within a GP as well, so that was great. But it was honestly so intimidating, me being there thinking “I've absolutely got no clue”. I could stick to the manual, and I guess it is manualised, but having a little bit more knowledge, a little bit something extra’ (university facilitator 3, APPLE-Tree study).

With experience, facilitators accepted that they did not need to be an expert or have extensive life experience, and that a ‘non-expert’ role could be beneficial.

‘Obviously we're not doctors, we're not scientists, but we have the information in front of us that we've based our research on’ (third-sector facilitator 3, APPLE-Tree study).

‘I feel like I've just learnt so much … I feel like I've got more confident as I've gone through having more experience … sometimes people might think that we're the experts and actually they are because they have so much more insight, so much more experience and they really know their relatives and themselves. If I do feel like sometimes people might say, what would you do, do you have any advice, and I would say that I'm here to facilitate the session for you and help you come up with strategies. So I think maybe not knowing all the answers, accepting I don't know all the answers and just being someone supportive and consistent is an important part of it’ (university facilitator 4, NIDUS-Family study).

Across group and one-to-one interventions, facilitators experienced the benefit of positioning clients as experts in control of their own health: this relieved perceived pressure and helped to develop confidence.

‘I felt like if someone had difficulties then it was my job to help them figure out those difficulties or come up with answers … As the groups progressed one of the things that was really, really nice was that the best answers came from the group themselves. So, I think having that confidence to just sit there and say nothing and just let people say what they want to say and let other people interrupt or give them suggestions was really beautiful and it was actually something that I learnt quite a lot of. That it's kind of better, that people don't always want answers. Sometimes people just want a space where they can talk and offload things and share things with other people’ (university facilitator 1, APPLE-Tree study).

Learning by experience and increasing confidence allowed facilitators to relax and enjoy the work. One facilitator found it ‘a really lovely thing to know that you've brought into someone's life and got them to think about things in a different way’ (university facilitator 4, APPLE-Tree study). Other facilitators began to describe sessions as ‘fun’ (third-sector facilitator 1, APPLE-Tree study), places they could ‘have a bit of a laugh’ with clients (university facilitator 1, NIDUS-Family study) and the ‘highlight of the week’ (university facilitator 2, APPLE-Tree study), even when facilitation was not going to plan:

‘They're an extraordinary bunch of people, and it was hard to keep them from not running away with the time …. Some of it wasn't pretty. There were several overruns of time, and [co-facilitator] and I, we would … well, “What are we doing wrong?” … it was our responsibility to manage the time, but it was never wasted time. … So, it was fun’ (NHS facilitator 2, APPLE-Tree study).

#### Subtheme 2: building clinical skills

Increasing confidence in the role of non-expert facilitator occurred alongside development of clinical skills. These were learnt through initial training, trial and error, and reflection in clinical supervision. Role-play was identified as the most helpful way of preparing, to decrease nervousness and uncertainty.

‘I know [clinical supervisor] loves to do role-play in supervisions and that's really helpful because it breaks down any kind of awkwardness that you might have with talking to other people, be that a co-worker or someone in the intervention’ (third-sector facilitator 3, APPLE-Tree study).

Some facilitators described how training could be ‘overwhelming’ (university facilitator 3, NIDUS-Family study) because although it could serve as preparation for ‘worst-case scenarios’ (university facilitator 3, APPLE-Tree study), discussing potential scenarios also increased worry about the possibility of being ‘out of their depth’. Across both interventions, when facilitators were observed by trainers and supervisors there was a fear of exposure of not having skills to manage difficult scenarios as well as clinical staff.

‘You do feel slightly underprepared purely because every session is different, and of course you can train for the first one, and [clinical supervisor] can ask you some tricky questions. But that did end up instilling a little bit of fear for anything else, and you're like, “Oh my God”’ (university facilitator 3, APPLE-Tree study).

Although facilitators recognised the need to develop clinical skills within and across intervention sessions, some experienced this as being ‘chucked in the deep end’ (university facilitator 3, NIDUS-Family study).

‘I feel like most of the learning was done throughout. A module would come up and I wouldn't really know what to do so I would bring it to supervision and ask for advice. So it was more learning as I went but at the beginning it definitely felt overwhelming, like, trying to know what modules to pick or how rigidly you stick to the modules and things like that’ (university facilitator 3, NIDUS-Family study).

As time progressed, facilitators reflected that despite the steep learning curve and associated anxieties, supervision built on learning in initial training.

‘[Clinical supervisor] gave really detailed feedback. So one of my typical ones the participant was quite rushed, they were a bit closed so [clinical supervisor] picked out points where I could have probed a bit more or there could have been a bit more of active listening or just simple things like just saying their name throughout sessions. Just things like that, really, just reinforcing what we learnt in training’ (university facilitator 5, NIDUS-Family study).

‘Although there's nothing to beat being thrown in at the deep end and having to deliver something. You learn to swim’ (third-sector facilitator 2, APPLE-Tree study).

Supervision was described by one facilitator as ‘a lifeline’ (university facilitator 5, APPLE-Tree study) and, by many, as a positive safe space to be supported, learn and share knowledge. It was seen to support ‘personal growth’ (third-sector facilitator 3, APPLE-Tree study) in clinical knowledge and skills.

‘I think the supervisors captured really well the spirit of adventure and the spirit of discovery, and, yeah, so they were very safe, entertaining, exciting. And, yeah, so that was important’ (NHS facilitator 2, APPLE-Tree study).

Facilitators described how supervision supported them to develop their facilitation style in areas such as being comfortable with silence (allowing clients space to think, reflect and say more); ‘soften[ing] the language’ used to elicit goals (third-sector facilitator 2, APPLE-Tree study) and discussing more sensitive topics in an organic way, less ‘like robots’ (third-sector facilitator 1, APPLE-Tree study); learning how to set ‘realistic’ goals using small steps to build bigger change (university facilitator 3, APPLE-Tree study); ‘being more flexible’ in approaching the material (third-sector facilitator 4, APPLE-Tree study) and seeing when something ‘can be used … creatively’ (university facilitator 1, APPLE-Tree study).

‘So that's how I started off and then I learnt in time that actually just having an organic conversation you can do that and listen to them and make them feel heard and not just trying to squeeze goals from them. So then I started really enjoying them and I've noticed that I was actually getting a lot better feedback from the [clients]’ (university facilitator 2, APPLE-Tree study).

Many facilitators commented on the flexibility of the content. Initially this caused worry, but with experience, the ability to tailor sessions was perceived to allow creativity and adapt material to be client-led, making it feel more meaningful.

‘There was enough structure for us to kind of go through the information but also enough flexibility for us to deliver it in our own way. So, me and [co-facilitator] can make it our own, and also, we can adapt it to each group individually’ (university facilitator 1, APPLE-Tree study).

‘It felt more personal to the dyad, it felt more individualised, and so it felt like you're making kind of a real difference, rather than just kind of reading through material’ (university facilitator 2, NIDUS-Family study).

#### Subtheme 3: the role of peer support

Facilitators noted the importance of a team environment that promoted good working relationships, peer support and open communication. This allowed facilitators to ‘have each other's backs if [they] had a problem’ (university facilitator 1, APPLE-Tree study):

‘So that's another thing that, of course, works well is having someone who can back you up, having someone who you can maybe have a bit of a moan to when things aren't going as planned. Because if [a participant is] complaining to you, that, in itself, is quite an isolating experience’ (university facilitator 5, APPLE-Tree study).

There was also a role for the wider team in being able to discuss concerns after sessions:

‘So everybody's always very responsive and terribly helpful. If you have a question, I've been very thrilled by the responsiveness of the team, can't fault it’ (third-sector facilitator 2, APPLE-Tree study).

Third-sector facilitators commented on peer support available during the work and through relationships forged in training and supervision:

‘I've enjoyed this absolutely enormously. I'm very impressed that the APPLE-Tree programme isn't ageist and it was prepared to take someone like me onboard, well past my sell-by-date, I'm sure in some people's opinion. So it was really lovely to be asked to provide this service, which I've thoroughly enjoyed doing, and working with such a variety of lovely people. It's been a great joy for me and probably good for my own well-being too. So I'd just like to say thank you to everyone’ (third-sector facilitator 2, APPLE-Tree study).

Peer support was fostered through group supervision, which helped overcome ‘the challenges of coming in from the outside and not being part of the team’ (third-sector facilitator 2, APPLE-Tree study).

‘I was a bit sceptical at first about group supervision I have to admit, because … I've never had it before it's always just been one on one I thought I'm not going to get enough time what if I'm struggling you know, but I have to say, [supervisor] was good at making sure we all got chance to speak and it was really useful listening to … [facilitator] and [facilitator] experience as well, because that could help me … which was really good … that really did work’ (university facilitator 1, NIDUS-Family study).

One facilitator did not find the group aspect of supervision helpful. They regarded supervision as a place to have questions answered, which individual supervision would have achieved more efficiently:

‘Actually, I didn't find supervision that helpful … It was helpful but when I talk about my dyads I know everything about them and I can ask my questions, but when other people are talking unless there is a very specific thing that could apply to other situations it doesn't really make the best use of time … I really sometimes struggled to focus on what was being said … I can remember that at the beginning I had lots of questions in supervision but after a while you just get into the rhythm and it just becomes less and less useful’ (university facilitator 6, NIDUS-Family study).

Peer support was possible despite working remotely, and was noted by many to help overcome difficulties that remote delivery brought (see subtheme 4):

‘When technology works it's brilliant, but when it doesn't it can be really difficult. You're trying to play a video and the video's buffering and your internet shuts out and suddenly you're kicked out of the session you're facilitating. And stuff like that does happen. So it's just about being calm and being in a good kind of facilitator duo where they can just take over and you calmly return to the [session]’ (university facilitator 2, APPLE-Tree study).

However, one facilitator discussed how working remotely meant colleagues could not see they were struggling to manage the workload or were feeling anxious before sessions:

‘So, I think the fact that we weren't all in the office meant just that pressure could build up’ (university facilitator 1, APPLE-Tree study).

#### Subtheme 4: remote delivery as a barrier to therapeutic relationships and confidence

Many facilitators described remote delivery as the hardest part of facilitation. Technology and internet connections had the potential to stop working: there could be a ‘lag … when people all start talking at the same time’ (university facilitator 2, APPLE-Tree study), the ‘microphone switches to whoever's making the most noise’ (NHS facilitator 2, APPLE-Tree study) and [you're left] ‘feeling bad that you sometimes have to mute people’ (university facilitator 3, APPLE-Tree study). Facilitators also worried how they would ‘come across’ online (NHS facilitator 2, APPLE-Tree study).

Facilitators found it harder to see ‘nonverbal cues as to whether somebody is … taken to certain material, so it was quite hard to get [clients] to open up’ (university facilitator 2, NIDUS-Family study).

Facilitators delivering group interventions found positive group dynamics were harder to foster online:

‘Working with such large groups of people remotely, technology came into quite a big factor. It's quite disruptive … we experienced earlier if someone can't log on fully and the conversation is getting kind of stunted or perhaps they're getting frustrated and they felt that they didn't want to do it because they were getting frustrated with technology. … just keeping such a large group of people remotely in check is quite difficult. I feel like it would be a lot different if you were in a room together face to face. So that was a big challenge’ (third-sector facilitator 3, APPLE-Tree study).

Facilitators saw greater potential for challenges arising from clients being in their own homes as opposed to a neutral space.

‘I think it makes it much harder – also your interpretation of people's body language is what you're going off of as well, and that's so different on Zoom than it is in person. People are also all in their own homes, so they're in their own environment, coming to the groups, as opposed to us all being in a room together, say, and working through the sessions. So, it definitely adds a bit more of a complex layer to it’ (university facilitator 4, APPLE-Tree study).

This was exacerbated for facilitators working with individuals with a diagnosis of dementia. As facilitators were not in the client's immediate environment, they were less able to identify external factors and ‘clues … that might be contributing’ to presenting challenges, such as patterned carpets increasing risk of falls (university facilitator 4, NIDUS-Family study). Facilitators also found it harder to communicate and easily engage clients who struggled more with technology, which on occasion led to miscommunication or tension. One facilitator described a client as ‘getting quite distressed by it’ (university facilitator 1, NIDUS-Family study).

One facilitator found that they overcame the barrier remote delivery posed to building therapeutic relationships by showing mistakes, which reinforced their non-expert position:

‘Showing the group that you can mess up and that you're just as clueless about these things as they are, so they kind of love that about it’ (university facilitator 1, APPLE-Tree study).

## Discussion

### Main findings

This study describes mostly positive experiences of non-clinically trained facilitators delivering remote psychosocial interventions to older adults. Gaps in life experience between clients and younger facilitators could create initial feelings of being ‘in at the deep end’ and fears of being ‘exposed’ as lacking expertise. These fears were allayed as facilitators learnt by experience, clients responded positively and facilitators saw in practice the benefit that a non-expert role could bring. This process of building confidence and experience was largely supported by training and supervision, where facilitators were supported to develop their facilitation and clinical skills. Peer support was mostly supportive in this process. There were specific challenges of delivering the intervention online as opposed to face to face, especially to forming therapeutic relationships.

Our findings align with previous studies that consider third-sector facilitator experience in delivering interventions to this population: Amador et al^10^ found that facilitators enjoyed the process despite initial concerns, and were able to use supervision and training to support feelings of capability; and Kingstone et al^12^ found that facilitators were able to use supervision to manage anxieties.

### Strengths and limitations

To our knowledge, this is the first study to consider the experiences of non-clinically trained facilitators working remotely with older adults with memory loss and their family carers that considers video call as well as one-to-one and group delivery.

As this facilitation work took place during the COVID-19 pandemic, this context probably influenced facilitator experience. Because the interviews were designed for the process evaluation, they did not specifically ask about effects outside the facilitator role; as such they were a more delimited, role-focused exploration of how facilitators experienced the programme.

### Implications

Our findings suggest that training and supervision for work with older adults might benefit from explicitly addressing the non-expert position and working across age differences, as some facilitators felt that developing therapeutic relationships with clients with decades more life experience was particularly daunting. Training for video call interventions might benefit from detailed coverage of technology-related scenarios, such as strategies when the internet cuts out, multiple people talking at once, decisions on muting clients and supporting clients unfamiliar with technology. In this context, training and supervision in groups and paired working increased feelings of support and connectedness despite remote work.

These findings are likely to be relevant in implementation of the NHS Long Term Plan.^5^ Understanding facilitators’ experiences of working with adults with memory loss, dementia and their family carers illuminated potential barriers to care that exist for older adults within the IAPT model.^5,8,9^ Moreover, findings suggest how the benefits of psychosocial interventions such as these might translate to wider community structures. It would be of interest to seek clients’ views on the same matter, as well as their experience of doing so via a remotely delivered intervention.

## Supporting information

Renouf et al. supplementary material 1 Renouf et al. supplementary material

Renouf et al. supplementary material 2 Renouf et al. supplementary material

## Data Availability

The materials that support the findings of this study, including the analytic code, are available on request from the corresponding author, P.R.
