# Supplementary material for: Non-clinically trained facilitators’ experiences of remote psychosocial interventions for older adults with memory loss and their family carers
Source: BJPsych Open. 2023 Oct 4;9(5):e174. doi: 10.1192/bjo.2023.558 (PMC10594201; doi:10.1192/bjo.2023.558)
Supplement: Renouf et al. supplementary material 1 — Renouf et al. supplementary material [file S2056472423005586sup001.docx]

**Appendix 1 – Topic Guide for APPLE-Tree**

**APPLE-Tree study**

**Facilitator interview Topic Guide**

**Introductions:** Thank you for agreeing to take part in this interview. As you know, I am recording it. I want to ask you about your experiences of delivering the APPLE-Tree intervention, and of attending training and supervision. We will use your suggestions to develop the training for facilitators delivering it in future. We would particularly welcome any thoughts about things that could be done differently and will value your honest opinion.

Please can you tell me which organisation you work for? How many series of group sessions have you delivered?

**Q. Tell us about your experience of delivering the APPLE-Tree intervention:**

- What do you think worked well?
- What were some of the challenges?
- What would you want to do differently?

**Prompts:**

- - *The initial set-up, Managing the group dynamics, Using zoom*
  - *Working with the other facilitator/ third helper/ UCL team*
  - *Delivering the difference components: diet, exercise, planning new activities, wellbeing, looking after your mental and physical health*
  - *The tea breaks*
  - *Sending in photos and other material*
  - *Goal calls and setting goals*
  - *Making plans to keep in touch with the group/ other participants after the main sessions have needed*

**Q. What was your experience of the training and supervision:**

- What was most useful?
- What would you change/improve?

**Q.** Can you tell us about a participant who you felt really benefited from the groups? And is there someone you worked with who you felt didn’t derive benefit? (prompt for details)

**Q. Before we finish, is there anything else you would like to add?**

**Thank you for your time and for taking part today.**
