# Supplementary material for: Non-clinically trained facilitators’ experiences of remote psychosocial interventions for older adults with memory loss and their family carers
Source: BJPsych Open. 2023 Oct 4;9(5):e174. doi: 10.1192/bjo.2023.558 (PMC10594201; doi:10.1192/bjo.2023.558)
Supplement: Renouf et al. supplementary material 2 — Renouf et al. supplementary material [file S2056472423005586sup002.docx]

**Appendix 2 – Topic Guide for NIDUS-Family**

Topic guide for facilitators

**Introduction:**

*“Hello, I am XXXX and my role is XXXX. Thank you for your time today, I wanted to find out more about your experiences in delivering the NIDUS-Family intervention. It would be interesting to explore things that you found went well and things that you feel could be improved to help us plan how this can be delivered in practice. There are no right or wrong answers, I’m interested in your experiences. This interview will be audio-recorded and transcribed, with your permission. Everything you say will be confidential and anonymised. Do you have any questions? If you are happy to continue, I will start recording.”.*

***Introduction***

**Q1. Can you tell me about the NIDUS-Family intervention?**

- What are your experiences of the programme so far?
- *Anything that was helpful?*
- *Were there times when you felt uncomfortable delivering it?*
- *Were there times the dyad felt uncomfortable receiving it?*

**Q2. Can you tell me about your training?**

- *How did you feel about delivering your first session?*
- *Can you give me an example…*
- Can you tell me what the top ‘take homes’ or ‘key messages’ you remember were from your training?
- Knowing what you do now, do you feel your training prepared you for your first session?
- Can you tell me about the support/ supervision you received through both training and when delivering?
- *Anything that was helpful – can you give me an example?*
- *What did you discuss with your supervision team – can you give me an example?*

***Values and approaches***

**Q3. Can you tell me more about your relationship with dyad XXX across the sessions?**

- *Do you feel you promoted the dyad to have choice (CA1.1)?*
- *Do you feel you promoted the dyad to have active agency? (CA1.2)*
- *Do you feel you built a sense of trust with the dyad? (CA1.2)*
- *Do you feel you actively listened?*
- *Do you feel your relationship provided mutuality and reciprocity? (CA1.2)*
- *Do you feel there was mutual respect between you and the dyad?*
- How do you feel your relationship affected the dyad attaining their goals?
- Do you feel the dyad had opportunities for meaningful engagement (able to actively participate, actively contribute ideas/ skills/abilities)?
- Can you tell me about the ‘role’ you played (parent-child relationship/ parent-parent relationship with dyad)

**Q4. Can you tell me about the dyads relationship?**

- *How did the dyad interact in the sessions?*
- *Who was involved in discussions?*
- *Who was involved in decision-making?*
- *Who took accountability for actions?*
- Expanding on the dyads relationship – Can you give me an example of where the PLWD led on a suggestion or idea?

***Goal attainment***

**Q5. Tell me about your experience of identifying issues/ needs with dyad XXXs…**

- *How did you discuss this?*
- *Who led this conversation?*
- *Who identified the issues?*
- *How did dyads interact?*
- *Did dyads agree?*
- How were dyad XXX goals related to – PLWD/Carer or both?
- After you identified the dyad XXX ‘needs’ tell me how you went about setting their goals…
- *If it is easier, talk me through a specific example.*
- *How did you link the need to their goal?*
- *How was the dyad involved in the discussion?*
- *Was it PLWD and carer? Tell me more…*

**Q6. Once the goals were set can you talk through how you developed [plans/ activities/ actions] for the dyad to work towards their goals…**

- How were the dyad involved?
- Tell me about a risk you discussed and how you managed this (when setting tasks/ goals)?
- [If relevant] Dyad XXX rated themselves as [+2/ 0,-1,-2] could you tell me your views on why?
- [If relevant] The outcome assessor [*you*] rated dyad XXX as [?] at 12-month follow up – do you agree? What would you have rated them?

***Strategies***

**Q7. Talk me through how the modules worked for dyad XXX?**

- What was your experience of delivering the manualised modules?
- How did you align dyad XXX goals to the modules?
- How do you feel the dyad XXX engaged with the modules?
- Can you give me an example where the modules helped motivate dyad XXX?
- Can you give me an example where the dyad didn’t understand or didn’t ‘click’ with the module?
- *[if relevant]* Did you have discussions around adapting their home?
- *[if relevant]* Tell me about how adaptations to the dyads home affected them achieving their goals?
- What are your views on having one facilitator for each dyad?

***Conclusion***

**Q. Before we finish, can I review the key points you mentioned about:**

- - **Goal attainment**
  - **Values and approaches**
  - **Strategies**
  - **Delivery**

**Q. Is there anything else you would like to add?**

**Q. Is there anything we haven’t covered you feel is important?**

**Thank you for your time and for taking part today.**
